# Climate change and continued professional development (CPD): Is it time for all CPD diaries to include carbon footprint estimates?

**DOI:** 10.1016/j.fhj.2025.100242

**Published:** 2025-03-20

**Authors:** Alexander Woywodt, Rebecca Kuruvilla, Sinead Stoneman

**Affiliations:** a Department of Renal Medicine, Lancashire Teaching Hospitals NHS Foundation Trust, Preston, London,UK; b Royal College of Physicians, UK; c Department of Nephrology, Cork University Hospital, Cork, Ireland

## Abstract

The triple threat of changing climate, loss of biodiversity and pollution poses a significant challenge to our patients and the planet, and healthcare contributes to all three elements of the threat. The carbon footprint of continued professional development (CPD) is increasingly recognised, although a cognitive dissonance exists whereby climate change is acknowledged but air travel to conferences continues unabated. A CO_2_ allowance for CPD activities has been suggested previously. We suggest that CO_2_ footprint estimates could be incorporated into existing CPD diaries as a step towards visualising the environmental impact of CPD. Electronic CPD diaries are already widely used and typically contain dates and locations for CPD activities. It would be relatively easy and inexpensive to add an estimate of CO_2_ footprint to these diaries. Such an approach would initiate reflection, promote insight and help facilitate behavioural change. We call on institutions involved in CPD licensing, administration and documentation to trial this approach and share their experience.

## Introduction

The threat of climate change to the planet, to the health of patients and to the provision of healthcare is incontrovertible.^1^ The threat is not confined to climate change alone, but also relates to loss of biodiversity and to pollution^2^ and the term triple threat has been used. It is important to acknowledge that reducing carbon footprint alone will not automatically preserve biodiversity or pollution. In nephrology, the carbon footprint of dialysis is well documented^3^ and other specialties have also started investigating their environmental impact, often focusing on waste reduction. In comparison, the carbon footprint of continued professional development (CPD) has only recently been acknowledged. Prompted by a recent congress of the European Renal Association (ERA), we focused on the carbon footprint of attending international congresses face to face^4^ and discussed ways to reduce the carbon footprint of such events. We proposed to prompt reflection by visualising carbon footprint estimates of in-person CPD. In this commentary, we suggest adding the carbon footprint of in-person CPD to existing CPD documentation, ie CPD diaries, registries and certificates. We acknowledge that the environmental footprint of such events is bigger than just air travel, as it involves accommodation, food, local travel and many other factors, but we feel that air travel to such conferences is a good starting point. We explore practicalities and opportunities, and consider potential barriers.

## The carbon footprint of CPD

There is increasing concern around climate change and efforts are currently under way across most medical specialties to reduce waste and make the delivery of care more sustainable. Examples include consumables in surgery,^5^ the reduction of volatile compounds in anaesthesia^6^ and the avoidance of disposable endoscopy equipment.^7^ The carbon footprint of CPD has only recently received detailed attention. We have previously attempted to delineate the carbon footprint of one well-established international meeting in our specialty, the 2023 European Renal Association congress. We estimated that air travel may have generated 5,808 tonnes of CO_2_, the equivalent of 356 sports utility vehicles (SUVs) circumnavigating the Earth. This CO_2_ footprint is also comparable with the annual CO_2_ emissions of 807 Europeans or the annual carbon footprint of 1,489 UK patients receiving in-centre haemodialysis – a phenomenal amount that will contribute to climate change not just in one short moment, but for years to come.^4^

The carbon footprint of conferences in many other specialties has been calculated and compared to virtual meetings. As an example, the carbon footprint of the 2019 in-person American Academy of Ophthalmology Congress with around 23,000 participants has been estimated at 39,910 tonnes of CO_2_.^8^ In comparison, the carbon footprint of the same congress delivered remotely in 2020 was 38 tonnes of CO_2_.^8^ Estimations of in-person conferences are imprecise as they assume the number of attendees, mode of transport and distance travelled. In addition, such estimates do not consider leisure travel onwards from the congress. Overall, it is worth emphasising that one cannot easily improve what is not being measured routinely^9^ and routine reporting of the carbon footprint should probably become the norm for congresses and perhaps also for smaller regional and local CPD workshops.

The carbon footprint of international conferences must be balanced against their irrefutable advantages. In-person discussion, serendipitous encounters as well as nurturing professional relationships are currently difficult to reproduce in virtual events. The advantages of in-person CPD and networking are beyond the scope of this opinion piece, but future technology may help to overcome some of the disadvantages of virtual meetings.^4^ Even the carbon footprint of a face-to-face event can be mitigated by a different location with an optimised carbon footprint, or multiple face-to-face meetings closer to participants’ homes.^8^ In addition, numerous steps can be taken to improve the sustainability of an in-person event itself, for example in terms of provision of food, local commute or waste.^10^

The next logical step in this direction, we believe, is to incorporate carbon footprints into CPD recording. Carbon footprints can be estimated for individual events, but healthcare providers often attend more than one congress annually and it would be more meaningful to estimate the carbon footprint generated by one individual in 1 year. Bostock^11^ proposed an annual CPD carbon allowance, and our proposal could be viewed as a step in that direction.

## How can we add a carbon footprint estimate to existing CPD documentation?

In many countries, such as Australia, Canada and the UK, CPD activities are already captured by electronic diaries. Similar mechanisms to record CPD exist elsewhere. Typically, these diaries feature a breakdown of activities by type, for example internal (departmental teaching), external (congress, workshop, etc.) and personal (reading text, writing, or refereeing manuscripts). In the UK, annual targets for CPD activities exist across most medical and surgical specialties and such targets are often used as a benchmark in annual appraisals.

For now, we propose an optional aspect of these CPD diaries capturing an estimate of carbon footprint for the travel to each CPD activity. Participants would state the mode of transport or virtual attendance when recording activities. Typically, the place of work is already registered in CPD diaries and the location of each CPD activity is also documented. We would suggest focusing on the commute to and from events because the remainder of the carbon footprint during a congress would be difficult to capture and cumbersome for individuals to record. In this sense, the estimate would represent a minimum estimate not including accommodation, food, urban commute or leisure during the event.

Validated algorithms to estimate carbon footprint for commute by plane, car or train already exist. The modified CPD app would then display the total travel carbon footprint to date throughout the year and a breakdown of CPD activities by mode of transport versus those activities attended remotely. It could also easily compare the current carbon footprint with that of the previous CPD period and feature, for example, a 5-year cycle with a trend over a longer period. A feature for reflection at the end of the year could also be part of this approach. An opt-out option should also be provided. As a next step, healthcare organisations could also suggest a carbon footprint target beneath which to obtain CPD points for the year’s activities, thereby allowing healthcare providers to manage their travel for conferences more efficiently. Individuals may even take up our suggestion before institutions adopt this approach and use commercially available solutions that calculate the carbon footprint of travel, such as Route Zero™. Such an approach may prompt individuals to reflect and thereby influence the decision to travel to conferences prior to the event. Speakers at events could also display their carbon footprint, for example with their conflict-of-interest statement, which is now required at many events. This would help to introduce a wider audience to the concept.

The approach we suggest would also encourage team efforts and cooperation. It could be helpful if teams could choose to share their individual and team carbon footprints, including trends over an extended period of time, for example, a 5-year period. Teams could also document if they agree with targets or a collective carbon footprint ‘budget’ for CPD activities. Similar approaches are often seen in online fundraising for charity, in which individual achievements are shared, including with the public, with aims and targets agreed by a fundraising team. Employers could consider a reward for teams that have reduced their CPD carbon footprint. However, Such a move would need to be considered carefully, as it should not be seen as invalidating the voluntary nature of our suggested approach.

## What are the barriers to our suggestions and how can they be addressed?

While our suggestion has numerous benefits, there are also challenges. Table 1 summarises potential barriers and how they can be addressed. The impact of attending congresses face to face is clearly more complex than just the carbon footprint of travel. Limiting the assessment to the commute to and from the conference venue is a gross underestimate of the overall footprint, which also includes the use of resources, consumption of food, pollution and waste, and loss of biodiversity. Long term, we should aim for a more comprehensive assessment of the environmental footprint of our congress attendance, but for now, we feel that the focus on travel is a reasonable first step.

**Table 1:** Potential barriers to our suggestions and suggestions to address them.

Barrier	Stakeholder	Comment/approach to mitigate
Estimates of CO_2_ footprint are imprecise and do not capture the complexity of the current triple crisis (climate change, biodiversity loss and pollution)	Institutions	Provide clarity regarding the methodology, acknowledge its limitations and emphasise the triple threat
Institutions using CPD diaries have various stakeholders, such as leadership, members and different types of healthcare providers, who have a spectrum of opinions on CPD and on the planetary crisis	Institutions	Proactively seek members and stakeholders’ opinions and discuss at meetings and conferences and seek to build consensus
Our suggestions may be a concern for organisations trying to make profit from conferences	Institutions	Organise open forum for conference coordinators and planners to explore ideas on how profit can be achieved in a virtual conference scenario
Institutions may have different expectations for various groups of healthcare providers	Institutions	Aim for consensus and harmonisation
Industry exhibitions generate income for conference organisers and any trend towards virtual CPD may jeopardise such income	Industry, traders	Develop virtual platforms for industry to interact with participants during online events
Perception of bureaucracy	Individual healthcare providers	User-friendly design of additional functionality in existing electronic CPD diaries
Perception of coercion and pressure	Individual healthcare providers	Make the scheme voluntary
Some countries may lack electronic documentation of CPD activities.	Individual healthcare providers	Develop a free app for individual CPD diaries which includes carbon footprint estimates. Add footprint of travel to paper-based records where electronic CPD diaries are unavailable
Change may be difficult to initiate and implement for individuals due to competing priorities and workload	Individual healthcare providers	Start with a voluntary pilot, capture feedback and share/publish results
Concerns about data protection	Individual healthcare providers	Existing CPD diaries are usually password protected. Take formal consent for including carbon footprint-related data. Location could be approximated without sharing home address

Another potential problem with our suggestion is that green CPD diaries could be perceived as another level of bureaucracy by healthcare providers, who already find the process of recording and reflecting on their CPD activities challenging and time-consuming. Asking them to record more data may be seen as an additional burden. In the worst case, our initiative might be viewed by some as another bureaucratic process that wastes time, leading to disengagement. The design of this additional CPD functionality will have to be user-friendly. Ideally, no further action would be required from existing users, other than opting in and consenting to the display of carbon footprint estimates as a one-off action. We suggest offering this functionality as a voluntary add-on to avoid the perception of coercion, although further worsening of the climate emergency may lead to a situation in which this becomes mandatory at institutional or even national level.

Similarly, it will be important to be balanced and open-minded about this approach and capture not only benefits, but also concerns. As an example, if healthcare providers are attending fewer in-person educational events in favour of more virtual educational activities, then our proposed approach may not capture the decreased opportunities for or the range of available activities. We would hope that this is something on which the individual could reflect on in their CPD diary and use to foster further debate within specialties or the wider profession.

A minor concern may relate to data protection and confidentiality, but existing CPD diaries are typically password protected already, and location data could be approximated to, for example, avoid sharing participants’ home addresses. Opponents may also emphasise the fact that carbon footprint data will only ever be estimates and it will be important to be open about this aspect and to be transparent regarding the methodology used to estimate carbon footprint.

Additional pitfalls could relate to the implementation of the approach proposed. All organisations involved in CPD have stakeholders with diverse perspectives and priorities. We propose that conferences should now provide more space to discuss how CPD can be delivered in the future, and we also feel that it is time to survey perceptions on this topic and seek views from all stakeholders. To overcome inertia and test our approach, we suggest small pilot trials involving enthusiastic individuals. Any experience with such pilots, both positive and negative, should be shared and published. It is also possible that different organisations in a country develop very different expectations for different groups of healthcare providers as to which level of face-to-face congress attendance is acceptable. In this scenario, we would aim for national consensus.

An interesting disadvantage of virtual meetings also exists around industry exhibitions, which provide organisers, venues and exhibitors with income. These stakeholders may be opposed to our approach, but we would suggest that equally interesting interactions between industry and participants should be possible in a virtual meeting. We feel that it is time for conference organisers to discuss this aspect of virtual meetings and explore new avenues for commercial success in a virtual congress scenario.

Our suggestion is focused on electronic diaries for CPD, which are not universally available. We feel that organisations in countries where CPD diaries are still paper-based should aim for electronic CPD diaries since they have advantages beyond just displaying carbon footprint data. Where this is not feasible, estimates of CO_2_ footprint for travel could be added to paper-based CPD diaries.

## Conclusion

A global health emergency has been declared, and health professionals have a key role in driving change.^2^ Many interventions to address the triple threat require discussion with multiple stakeholders, policy change and often considerable investment. In comparison, changing our CPD habits is a personal decision that we can all make today and one that does not involve approval from others, policy change or large-scale investment. There is cognitive dissonance within the medical and scientific community in which the planetary crisis is widely acknowledged, yet the rebound of air travel to conferences after the COVID pandemic is accepted or even welcomed. CPD may only constitute a small percentage of the overall environmental footprint of healthcare and reducing carbon footprint alone will not suffice to address the current planetary crisis. A reduction of CPD-related carbon footprint could be an excellent starting point, which may lead to wider action. Visualising the carbon footprint of our own CPD habits may thus become a crucial step towards a wider reflection of the environmental footprints of our professional activities. The corporate world, retail and even personal banking have begun to be transparent about the environmental footprint of their goods and services, which is likely a crucial step in visualising the environmental footprint of our daily lives. If it is becoming standard practice to visualise the environmental footprint of an online purchase and its delivery as part of our private lives, then the effects on climate, pollution and biodiversity of our CPD activities should be equally visible. Our approach could also serve as an important tool against climate change fatigue and defeatism. A conscious decision to reduce our aeroplane travel to congresses abroad could also lead to a wider reassessment of travel habits for holiday and leisure and of our daily work commute. The technical implementation should not be difficult nor costly, particularly if electronic CPD diaries already exist. There are additional benefits of hybrid/virtual conferences for low-income countries, where many healthcare providers cannot afford to attend events abroad in person.^12^ Now is the time to address the current climate and nature crises as a global health emergency and to act on the carbon footprint of our CPD activities. We call on healthcare institutions that govern CPD diaries to pilot this approach and share results.

## Funding

This research did not receive any specific grant from funding agencies in the public, commercial, or not-for-profit sectors.

## CRediT authorship contribution statement

**Alexander Woywodt:** Writing – review & editing, Writing – original draft, Supervision, Project administration, Conceptualization. **Rebecca Kuruvilla:** Writing – review & editing, Conceptualization. **Sinead Stoneman:** Writing – review & editing, Writing – original draft, Conceptualization.

## Declaration of competing interest

The authors declare that they have no known competing financial interests or personal relationships that could have appeared to influence the work reported in this paper.
